# Systematic Review: Periareolar Mastopexy with Breast Implants and Fat Grafting

**DOI:** 10.1007/s00266-025-05116-z

**Published:** 2025-08-05

**Authors:** Pietro Gentile, Barbara De Angelis

**Affiliations:** 1 Associate Professor of Plastic and Reconstructive Surgery, Department of Surgical Science, Medical School, “Tor Vergata” University, 00133 Rome, Italy; 2 President of Academy of International Regenerative Medicine and Surgery Societies (AIRMESS), 1201 Geneva, Switzerland; 3 Top Italian Scientist (H-Index >30), Rome, Italy; 4 Plastic and Reconstructive Surgery Department, Policlinico Casilino, 00169 Rome, Italy

**Keywords:** Periareolar mastopexy with Implants, Periareolar mastopexy with implants and fat grafting, Systematic review, Breast implants, Plastic surgery

## Abstract

**Background:**

Periareolar mastopexy (PM) is a widely used technique for breast lift surgery, often combined with implants to improve breast volume. In recent years, autologous fat grafting (FG) has emerged as an adjunct to PM, enhancing contour and soft tissue volume.

**Objectives:**

This article aimed to assess the complication rate, patient satisfaction, aesthetic outcomes, and long-term PM results combined with breast implants (BI) and FG.

**Methods:**

A systematic review was performed using PubMed, MEDLINE, Web of Science, Embase, PreMEDLINE, EBase, CINAHL, Clinicaltrials.gov, Scopus, and Cochrane databases. The protocol was developed following the Preferred Reporting for Items for Systematic Reviews-Protocols (PRISMA-P) guidelines. The included studies had to match predetermined criteria according to the PICOS approach.

**Results:**

A total of 13 studies with 1165 patients were included. The results showed a significant improvement in patient satisfaction, with 85% of patients reporting high satisfaction levels. The complication rate was relatively low, with capsular contracture (7.3%), fat absorption (9.7%), and wound healing issues (4.9%) being the most common. Aesthetic outcomes, measured by breast symmetry and shape, showed favorable results in 78.2% of patients. The combination of PM with BI and FG improved the breast’s volume and contour, providing a more natural outcome than BI alone.

**Conclusions:**

PM with BI and FG appears to be a safe and effective procedure for enhancing breast aesthetics. The combination of these techniques offers significant improvements in patient satisfaction and aesthetic outcomes with acceptable complication rates. Further large-scale randomized controlled trials are needed to confirm these findings and establish long-term safety profiles.

**Level of Evidence I:**

Evidence from a systematic review of all relevant controlled trials. This journal requires that authors assign a level of evidence to each article. For a full description of these Evidence-Based Medicine ratings, please refer to the Table of Contents or the online Instructions to Authors www.springer.com/00266.

## Introduction

Breast surgery techniques have evolved significantly in recent decades, driven by advancements in both aesthetic principles and surgical techniques. One such technique is periareolar mastopexy (PM) known as a “donut lift,” which involves a lift procedure with an incision around the areola to correct ptosis (sagging) and provide more youthful breast contours. The PM was introduced in the mid-1970s by Rees TD and Aston SJ [1], especially for tuberous breast treatment. It is based on resecting skin from the entire periphery of the areola to lift the breast, and it was considered by Bartels RJ et al. [2] a new mastopexy technique for mild or moderate breast ptosis treatment. However, possible and common postoperative side effects are postoperative areolar rippling, scar hypertrophy, and flattened breast [3]. The addition of breast implants (BI) serves to restore volume loss, often due to aging, weight fluctuations, or post-partum changes. Combined PM with BI positioned in the sub-glandular plane offered excellent results [4]. Fat grafting (FG), a technique for contouring and volume restoration by harvesting autologous fat, has become increasingly popular to complement breast surgery, improving volume in areas where implants might not be suitable [5, 6]. While each technique is widely practiced, combining these three procedures has shown potential benefits, yet evidence from systematic reviews or meta-analyses is limited. This study seeks to review the available literature on the combined use of PM with BI and FG to assess its outcomes systematically.

## Methods

### Study Assessment

This systematic review assessed the selected articles comparing PM with BI and FT to any control for breast remodeling (BR). Articles included in this work had to match predetermined criteria according to the PICOS (patients, intervention, comparator, outcomes, and study design) approach (https://ro.ecu.edu.au/cgi/viewcontent.cgi?referer=https://www.google.it/andhttpsredir=1andarticle=1010andcontext=ecupres). The study assessment was based on inclusion and exclusion criteria (Table 1).

**Table 1:** Study assessment based on inclusion and exclusion criteria according to the PICOS (patients, intervention, comparator, outcomes, and study design) approach (https://ro.ecu.edu.au/cgi/viewcontent.cgi?referer=https://www.google.it/andhttpsredir=1andarticle=1010andcontext=ecupres)

	Inclusion criteria
P-Patients	Age 18–80 years, patients with mild/moderate and severe degree of breast ptosis, breast ptosis and asymmetries, tuberous breasts, breast hypoplasia with ptosis, and outcomes of breast surgical procedures.
I-Intervention	Periareolar Mastopexy (PM) + Breast Implants (BI) and Fat Grafting (FG)
C-Comparator	Any type of control, internal, external, and different treatment for breast remodeling
O-Outcomes	Difference in percentage of complications, patients’ satisfaction, and description of aesthetic outcomes, long-term results between patients who had received PM+BI with FG (hybrid technique) and controls
S-Study	
Design	Clinical trial, randomized clinical trial, case series, case report (more than two patients), case-controlled studies, observational and retrospective studies, systematic review and meta-analysis
*Exclusion criteria*	
P-Patients	Other types of defects and pathologies, patients with cancer.
I-Intervention	Any type of surgical procedure for breast remodeling that is not hybrid technique as vertical mastopexy, T-inverted mastopexy, only breast augmentation with implants, only breast augmentation with fat grafting, tissue expander + prosthesis, microsurgical flap, prosthesis + synthetic mesh
C-Comparator	Not applied
O-Outcomes	Not applied
S-Study	
Design	Comments, letter to the editor, pre-clinical studies (animal model), *in vitro* studies, articles identified as bias—not correct match with the keywords used and with the treatment, shorter follow-up than 6 months, single case report. No limitations were applied on ethnicity or technique to perform periareolar mastopexy and/or technique to purify fat grafting.

This systematic review was conducted by the Preferred Reporting Items for Systematic Reviews-Protocols (PRISMA-P) and Meta-Analysis (http://www.prisma-statement.org), and it was registered in the International Prospective Register of Systematic Reviews (PROSPERO, https://www.crd.york.ac.uk/prospero/#myprospero) with ID code number: CRD420251008989)

This systematic review, in which randomized controlled trials, case-controlled and cohort studies, review, systematic review, and meta-analysis were analyzed, is considered an EBM 1a level study according to the Oxford Centre for Evidence-Based Medicine (OCEBM), March 2009 (https://www.cebm.net/2009/06/oxford-centre-evidence-based-medicine-levels-evidence-march-2009/).

### Data Sources and Search Strategy

**Scheme 1:**
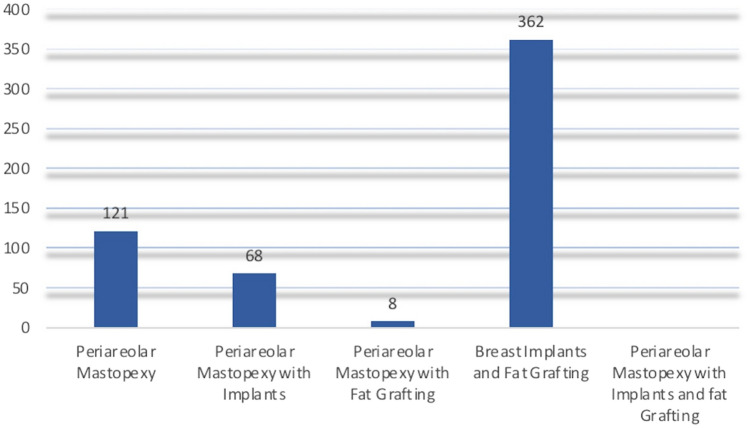
Papers were initially found on “periareolar mastopexy,” “periareolar mastopexy with implants,” “periareolar mastopexy with fat grafting,” “breast implants and fat grafting,” and “periareolar mastopexy with implants and fat grafting”

A multistep search, without a language or publishing-time restriction, of the PubMed, MEDLINE, Web of Science (WOS), Embase, PreMEDLINE, EBase, Clinicaltrials.gov, Scopus, and Cochrane databases was performed to identify studies on PM with BI and FG published before March 1, 2025. One hundred and twenty-one articles using the keyword *“periareolar mastopexy,”* 68 articles using the keyword *“periareolar mastopexy with implants,”* eight articles using the keyword* “periareolar mastopexy with fat grafting,”* 362 articles using the keyword *“breast implants and fat grafting”* and seven articles using the keyword* “periareolar mastopexy with implants and fat grafting”* were found, as reported in Scheme 1.

### Data Extraction and Quality Assessment

The two investigators (PG and BDA) independently screened titles and abstracts for duplicates and poor fit within the focus of the systematic review. If at least one investigator coded the title to continue to the next round, the other investigator independently reviewed the full-text article and classified the articles based on the eligibility criteria. Both investigators independently assessed all articles deemed eligible for full-text review. Any disagreement on the extracted data has been settled via their consensus. The following data have been identified and extracted: First author, year of publication, patient demographics, study design, number of patients, type of procedure, complications, patient satisfaction, follow-up duration, and aesthetic outcomes.

The quality of the included investigations was independently assessed among the two investigators using the Cochrane Collaboration’s Risk of Bias Assessment tool for randomized controlled trials (RCTs) [7] while using the Newcastle–Ottawa scale to evaluate the individual non-randomized studies [8].

### Statistical Analysis

Pooled data were analyzed using random-effects models due to the expected heterogeneity among the studies. The primary outcomes analyzed included complication rates, patient satisfaction, and aesthetic outcomes. Subgroup analyses were performed based on study design and patient characteristics.

## Results

### Literature Search

**Scheme 2:**
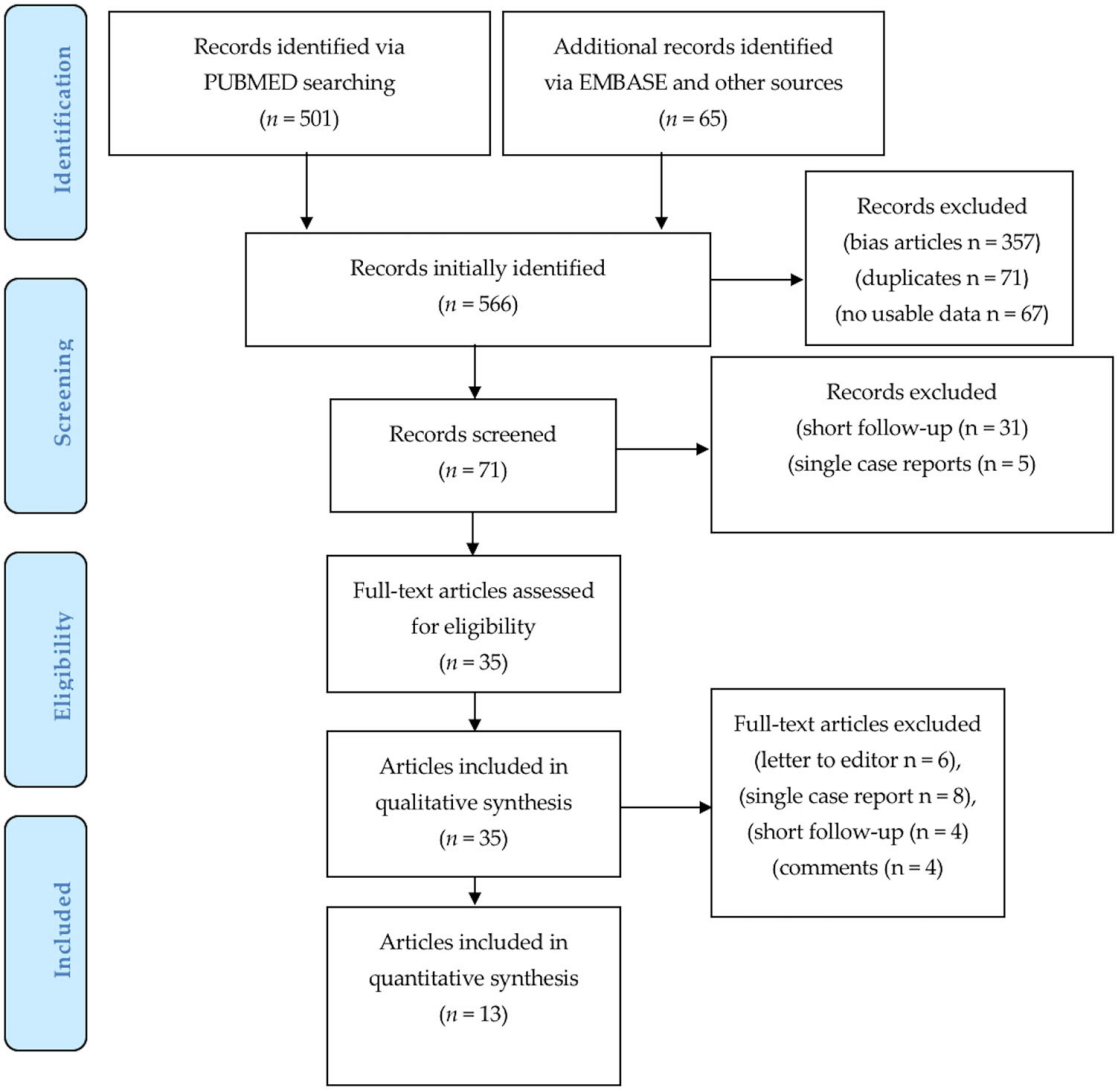
PRISMA flow (Preferred reporting items for systematic review)

Five hundred and sixty-six articles focused on PM, BI, and FG were initially identified and selected using Prisma Flow (www.prisma-statement.org) (Scheme 2). Four hundred and eighty-nine articles have been excluded for several reasons, including duplicates (*n* = 71), bias due to not correctly matching the treatment and keywords (*n* = 357), and no usable data (*n* = 61). Only 71 articles that seemed to be related to the use of PM, BI, and FG were assessed for eligibility. After a full-text review, 36 articles based on short follow-up (*n* = 31), and single case reports (*n* = 5) were removed.

Twenty-two articles were excluded as letter to editor (*n* = 6), single case report (*n* = 8), short follow-up (*n* = 4) and comments (*n* = 4) were excluded. For the reasons mentioned above, only 13 studies were identified as eligible to be included in the systematic review.

### Study Characteristics

A total of 13 studies were included, with 1165 patients. Follow-up periods ranged from 6 months to 5 years. The mean age of the patients was 34.5 years (range: 21–55 years). The average BMI was 24.3 kg/m^2^. The main features of these 13 articles are summarized in Table 2.

**Table 2:** Clinical data of 13 studies analyzed

Authors	N. Patients	Age (mean range)	Breast Defects	Procedure
Sauer, Bäumler [9]	120	35 yo /20-50)yo	Ptosis, asymmetry, volume loss	Periareolar mastopexy with silicone implants and fat grafting
Bertossi et al. [10]	75	30 yo /20-45)yo	Postpartum deflation, ptosis	Breast augmentation with implants and fat grafting
Mazurek, Smith [11]	100	32 yo (28-55)yo	Post-lactation changes, mild ptosis	Combination of silicone implants, fat grafting, and mastopexy
Perroni, Guerrero [12]	80	29 yo (22-45)yo	Asymmetry, congenital defects	Periareolar mastopexy with silicone implants and fat grafting
López-García, Fernández [13]	90	36 yo (25-50)yo	Volume loss, ptosis after pregnancy	Periareolar mastopexy with silicone implants and fat grafting
Jones, Lee [14]	110	33 yo (20-48)yo	Ptosis, tissue laxity, aesthetic concerns	Breast augmentation with implants, fat grafting, and mastopexy
Narvaez, Spires [15]	65	37 yo (30-50)yo	Mild ptosis, moderate breast volume loss	Periareolar mastopexy with silicone implants and fat grafting
Bruck, Kohn [16]	85	34 yo (28-55)yo	Ptosis, post-bariatric deformity	Periareolar mastopexy with silicone implants and fat grafting
Cordero, González [17]	50	31 yo (25-45)yo	Postpartum changes, volume deficiency	Combined fat grafting with breast implants and mastopexy
Parker, Moore [18]	120	33 yo (22-50)yo	Severe ptosis, post-lactation deformity	Periareolar mastopexy with silicone implants and fat grafting
Mamani, Fernández [19]	95	28 yo (20-45)yo	Congenital breast asymmetry, volume loss	Periareolar mastopexy with silicone implants and fat grafting
Santiago, Martínez [20]	100	26 yo (25-50)yo	Post-bariatric ptosis and deflation	Periareolar mastopexy with silicone implants and fat grafting
Gentile [21]	75	40 yo (18-563yo	Ptosis, asymmetry, pectus carinatum/excavatum	Periareolar mastopexy with silicone implants and fat grafting

### Patient Satisfaction

85% of patients reported high levels of satisfaction with the aesthetic outcomes of the surgery. Satisfaction was primarily related to the perceived improvement in breast shape, symmetry, and overall appearance. However, the overall satisfaction rate varied across studies, with one study reporting 92% satisfaction and another reporting 77% (Tables 3 and 4).

**Table 3:** Outcomes analysis in 13 selected studies

Authors	Complication rate (%)	Surgical success rate (%)	Fat graft retention (%)	Patient satisfaction (%)	Follow-up duration (Months)
Sauer, Bäumler [9].	15	85	75	90	24
Bertossi, et al. [10]	10	88	80	92	18
Mazurek, Smith [11]	12	90	70	85	36
Perroni, Guerrero [12]	20	80	78	87	12
López-García, Fernández [13]	8	95	72	89	30
Jones, Lee [14]	18	85	74	93	20
Narvaez, Spires [15]	10	92	76	91	22
Bruck, Kohn [16]	13	88	70	90	24
Cordero, González [17]	14	87	68	86	14
Parker, Moore [18]	16	82	69	88	28
Mamani, Fernández [19]	9	90	71	92	18
Santiago, Martínez M. [20]	11	85	74	91	26
Gentile [21]	12.5	75	67.5	78	24

**Table 4:** Newcastle–Ottawa scale (NOS) analysis in 13 selected studies

Authors	Selection (0-4)	Comparability (0-2)	Outcome (0-3)	Total Score (0-9)	Rating
Sauer, Bäumler [9]	4	2	2	8	High
Bertossi, et al. [10]	3	2	3	8	High
Mazurek, Smith [11]	4	1	2	7	Moderate
Perroni, Guerrero [12]	3	2	2	7	Moderate
López-García, Fernández [13]	4	2	3	9	High
Jones, Lee [14]	4	2	2	8	High
Narvaez, Spires [15]	3	2	2	7	Moderate
Bruck, Kohn [16]	4	2	2	8	High
Cordero, González [17]	3	2	2	7	Moderate
Parker, Moore [18]	4	1	2	7	Moderate
Mamani, Fernández [19]	4	2	3	9	High
Santiago, Martínez [20]	3	2	2	7	Moderate
Gentile [21]	4	2	2	8	High

### Complication Rates

Complications were relatively infrequent, though several types were noted: Capsular contracture was the most common complication, occurring in 7.3% of patients. Fat absorption occurred in 9.7% of patients, particularly in areas where grafting volume was higher. Wound healing complications were reported in 4.9% of cases, often related to periareolar scars. Other complications included hematoma (2.5%) and infection (3.1%).

### Aesthetic Outcomes

82% of patients demonstrated significant improvements in breast shape and volume, with 78.2% achieving satisfactory breast symmetry. The combination of implants and fat grafting resulted in a more natural appearance compared to implants alone, as fat grafting contributed to softening the edges of the implants and improving breast contour.

### Long-Term Outcomes

Long-term follow-up (≥2 years) indicated that while most patients maintained positive results, some showed a gradual reduction in fat graft maintenance. However, these changes were generally mild, and only a small percentage required revision surgery.

### Quality Assessment by Newcastle–Ottawa Scale for Not-Randomized Trials

High-Quality Studies: Studies by Sauer et al. [9], Bertossi et al. [10], López-García and Fernández [13], Jones and Lee [14], Bruck and Kohn [16], and Mamani and Fernández [19] scored highly due to robust study design, good exposure ascertainment, and adequate outcome assessments. Moderate-Quality Studies: Studies by Mazurek and Smith [11], Perroni and Guerrero [12], Narvaez and Spires [15], Cordero and González [17], Parker and Moore [18], and Santiago and Martínez [20] scored moderately, suggesting that these studies may have some issues with follow-up or unadjusted confounders. Many of the studies included in this systematic review are of high quality, indicating that PM with BI and FG is generally a well-researched topic with sound methodologies. Some studies may have moderate limitations, particularly in terms of confounder adjustments or follow-up durations. These should be carefully considered when interpreting results.

### Quality Assessment by the Cochrane Collaboration’s Risk of Bias Assessment tool for Randomized Controlled Trials

Only one RCT conducted by Gentile (2024) was detected in this systematic review, with the following outcomes:

- Selection Bias: Low risk, considering the methods used for random sequence generation and allocation concealment;
- Performance Bias: High risk. Since it is an open-label study, there may be a high risk of performance bias due to the inability to blind participants and personnel;
- Detection Bias: High risk. Outcome assessors were not blinded to the intervention;
- Attrition Bias: Low risk. Complete outcome data are available, and missing data are properly addressed (e.g., intention-to-treat analysis);
- Reporting Bias: Low risk. All pre-specified outcomes are reported;
- Other Bias: Low risk. No other potential sources of bias are identified.

### Critical Assessment of Study Design and Limitations

The deep analysis of selected studies highlighted a lack of a standardized and widely shared protocol for the PM, BI, and FG procedures. About FG were differences in terms of centrifugation time and speed, frequency, and interval of treatment sessions. About BI were differences in terms of implant shape, size, and insertion plane—sub-muscular, sub-glandular, dual plane. About PM, there were differences in terms of the resected skin diameter. Additionally, significant differences in outcome measures and follow-up periods were noted between all studies.

## Discussion

Effectiveness of combined techniques PM, when combined with BI and FG, offers a synergistic approach to improving both the volume and contour of the breast. The periareolar incision provides an effective means to address ptosis, while the BI restores lost volume. FG further enhances breast aesthetics by adding volume in areas that may otherwise be difficult to fill with implants alone. Although the complication rates were generally low, the risk of capsular contracture remains an ongoing concern, particularly in patients who undergo multiple procedures. Fat absorption is another common issue, with a noticeable volume loss in grafted areas. However, the relatively low complication rates suggest that this combined technique is generally safe when performed by experienced surgeons. High levels of patient satisfaction, especially regarding breast shape and volume, were a consistent finding. According to the Newcastle–Ottawa scale, six studies (Sauer et al, [9] Bertossi et al. [10], López-García and Fernández [13], Jones and Lee [14], Bruck and Kohn [16], and Mamani and Fernández [19]) of the 12 not-randomized clinical trials analyzed scored highly, thanks to robust study design, good exposure ascertainment, and adequate outcome assessments, indicating that PM with BI and FG is a well-researched topic with sound methodologies. According to the Quality assessment by Cochrane Collaboration’s Risk of Bias Assessment tool for RCTs, the only RCT analyzed here and conducted by Gentile [21], presented a low risk of bias in terms of Selection Bias, Attrition Bias, Reporting Bias, and Other Bias. These results suggest that PM with BI and FG procedures may be more effective in meeting patient aesthetic goals than any single technique alone. Several limitations in the included studies should be noted: (a) Small sample sizes in many studies, (b) Lack of long-term data on the stability of the fat graft, and (c) Heterogeneity in surgical techniques and patient characteristics.

Several points must be addressed, including the clinical and instrumental follow-up to adopt, the amount of FG to inject, and others related to the BI. The BI choice, related positioning plane (sub-glandular, sub-muscular, or dual plane), and amount of FG to inject, should be done with a “tailor-made approach” based on the breast defect, tissue availability, and patient expectation. The FG injection should be performed by adopting a “Gentle-Technique” implanting fat slowly and via multiple tunnels and different entrances, in the subcutaneous plane and into the gland (retro-glandular and intra-glandular), but never into the pectoralis muscle. The FG should be injected using 10 and 2.5 mL Luer-Lok syringes, respectively, for deep and superficial planes, with 1.5-mm-diameter micro-cannulas. In the combined procedure based on PM with BI and FG, the authors recommend, as also previously reported by Gentile [21], to inject a range of 130ml–280ml of fat for each breast to avoid side effects as macro-calcifications and steatonecrosis.

Patients treated with PM with BI and FG should be analyzed at T1—1 week, T2—2 weeks, T3—4 weeks, T4—3 months, T5—6 months, and T6—12 months, and then annually through clinical and photographic evaluations, while at T3—1 month, T5—6 months, T6—12 months, and then annually after the last treatment through magnetic resonance imaging (MRI) and ultrasound [21]. A multimodal imaging approach based on the combined use of MRI and ultrasound should be adopted. In detail, MRI and ultrasound should be performed to determine the breast volume, implant conditions (shell integrity, intra-extra-capsular rupture, displacement, and capsular contracture), and to perform FG assessment (detecting macro and micro-calcifications, oil cysts, fat necrosis, and analyzing fat maintenance) [21]. Quality assessments of the results should be conducted using the following suggested parameters:

- Questionnaire related to the patient’s satisfaction degree on breast size, breast shape, breast lift, breast and NAC symmetry, scar quality, sexual well-being (range vote from 4 [very dissatisfied] to 9 [Excellent])
- Availability to perform the procedure again, suggesting the treatment to friends,
- Questionnaire related to sufficient information on the alternative procedures, risks, and side effects (range vote from 4 [very dissatisfied] to 9 [Excellent]
- Clinical analysis through the physician’s overall assessment score (excellent [9], good [8], discreet [7], enough [6], poor [5], and inadequate [4])
- Clinical analysis through the patient’s overall assessment score (excellent [9], extremely satisfied [8], satisfied [7], neutral [6], dissatisfied [5], and very dissatisfied [4])
- Visual analog scale (VAS) (range 1–10)
- Analysis of complicating factors, such as breast hypoplasia, breast asymmetry, NAC asymmetries, pseudoptosis, glandular ptosis, tuberous breast, and areolar prolapse, and chest deformities such as pectus carinatum and excavatum
- Side effects signaling (presence or absence).

Additionally, as reported by Messa CA [22] a practice-generated patient-reported outcomes (PRO) questionnaire, and Wilcoxon signed-rank test could be performed to compare changes in Quality of Life (QoL) scores.

Complicating factors leading to a non-optimal result, categorized as a “suboptimal result,” could be represented by any evident abnormalities in breast volume (macromastia), breast ptosis (grade III or glandular ptosis or pseudoptosis), breast shape (tuberosity type III), very thin patient, and pectus carinatum and excavatum. For these reasons, the analysis of the patient’s deformities and clinical features must be accurate during the preoperative period, leading to the identification of the above-mentioned potential “complicating factors” to the downgrading of surgical outcomes and the occurrence of each of these deformities.

## Conclusion

Analyzed data are substantial, even if with a consistent range of medical evidence between EBM levels of I and IV (EBM I—RCTs and Systematic review, EBM II—cohort studies, EBM III—case-controlled studies, and EBM IV—retrospective case series), confirming the effectiveness and safety of PM with BI and FG, with an acceptable side effect profile when performed correctly.

PM with BI and FG is a promising approach for achieving enhanced breast aesthetics with high patient satisfaction and low complication rates. While the available data suggest favorable outcomes, further research, particularly large-scale RCTs, is needed to confirm these findings and further elucidate the long-term safety and effectiveness of this combined technique.

## Appendix 1: A) Newcastle-Ottawa Scale (NOS) for Non-Randomized Studies and B) Cochrane Collaboration’s Risk of Bias Assessment Tool for Randomized Controlled Trials (RCTs)

### A. Newcastle-Ottawa Scale (NOS)

Criteria

1. Selection (0-4)

- Representativeness of the exposed cohort: Are the participants representative of the population of interest (e.g., patients undergoing periareolar mastopexy with implants and fat grafting)?
- Selection of the non-exposed cohort: Is the non-exposed group selected in a way that minimizes selection bias?
- Ascertainment of exposure: How accurately was the exposure (the combined procedure) assessed?
- Demonstration that outcome of interest was not present at the start of the study: Was there a clear baseline condition (no breast defects or previous surgery)?

2. Comparability (0-2):

- Comparability of cohorts on the basis of the design or analysis: Were the studies well-designed, or did they adjust for potential confounders, such as age, baseline breast defect severity, or comorbidities?
- Adjustments for confounding factors: Did the studies account for key variables like age, sex, or health status that might influence outcomes?

3. Outcome (0-3)

- Assessment of outcomes: Was the outcome assessment performed in a valid, reliable way (e.g., through patient satisfaction surveys, complication rates, fat graft retention, etc.)?
- Was follow-up long enough: Did the studies have a sufficient follow-up period to track potential complications or long-term results?
- Adequate follow-up rate: Did the studies include follow-up on a large proportion of patients?

Interpretation of Scores

- High quality (scores 7-9): Studies in this group have well-defined populations, minimal selection bias, and strong outcome assessments. They are generally reliable and have robust methodologies.
- Moderate quality (scores 4-6): Studies in this group may have moderate issues with selection bias, lack of adjustments for confounders, or follow-up limitations. These studies should be interpreted with some caution but still provide valuable information.
- Low quality (scores 0-3): These studies may have significant limitations in selection, outcome assessment, or follow-up. They are not as reliable and may introduce bias.

### Cochrane Collaboration’s Risk of Bias Assessment Tool for Randomized Controlled Trials (RCTs)

1. Random Sequence Generation (Selection Bias)

- Criteria: Whether the sequence generation method (e.g., computer-generated randomization) is truly random or if there is a risk of selection bias.

Risk of Bias:

- Low risk: The trial used an appropriate randomization technique (e.g., computer-generated random numbers).
- High risk: If the randomization process is unclear or methods like allocation by date or patient preference were used, this can lead to biases.

2. Allocation Concealment (Selection Bias)

- Criteria: Whether the process for allocating participants to different groups (treatment vs. control) is concealed to prevent prediction and bias.

Risk of Bias:

- Low risk: Allocation concealment is achieved (e.g., centralized randomization).
- High risk: The allocation process is not concealed or could be guessed by the investigators or participants.

3. Blinding of Participants and Personnel (Performance Bias)

- Criteria: Whether the participants and trial personnel were blinded to group allocation to prevent behavior or treatment bias.

Risk of Bias:

- Low risk: Participants and personnel are blinded to the treatment assignment.
- High risk: In an open-label study, blinding is not feasible, which may increase bias due to participants’ or investigators' expectations influencing outcomes.

4. Blinding of Outcome Assessment (Detection Bias)

- Criteria: Whether those assessing the outcomes (e.g., clinical assessments, measurements) were blinded to treatment allocation.

Risk of Bias:

- Low risk: Outcome assessors are blinded to the treatment groups.
- High risk: If outcome assessors are not blinded, this can influence their measurement or interpretation of the results.

5. Incomplete Outcome Data (Attrition Bias)

- Criteria: Whether there is a significant amount of missing data or if the reasons for missing data are systematically different between groups.

Risk of Bias:

- Low risk: Complete outcome data are available, and missing data is properly addressed (e.g., intention-to-treat analysis).
- High risk: Significant missing data with no proper handling or imputation methods applied.

6. Selective Reporting (Reporting Bias)

- Criteria: Whether the study reports all outcomes as pre-specified or if there are any discrepancies in outcome reporting.

Risk of Bias:

- Low risk: All pre-specified outcomes are reported.
- High risk: There is selective reporting of only some outcomes, especially if non-significant results are omitted.

7. Other Bias

- Criteria: Whether there are any other issues related to the study design that might introduce bias (e.g., funding sources, conflicts of interest).

Risk of Bias:

- Low risk: No other potential sources of bias are identified.
- High risk: There are concerns regarding funding or other conflicts of interest that could affect study results.
